# Candidemia in the elderly: What does it change?

**DOI:** 10.1371/journal.pone.0176576

**Published:** 2017-05-11

**Authors:** Francesco Barchiesi, Elena Orsetti, Sara Mazzanti, Francesca Trave, Aldo Salvi, Cinzia Nitti, Esther Manso

**Affiliations:** 1 Dipartimento di Scienze Biomediche e Sanità Pubblica, Clinica Malattie Infettive, Università Politecnica delle Marche, Ancona, Italy; 2 Medicina Internistica e Subintensiva, Azienda Ospedaliero Universitaria, Ospedali Riuniti Umberto I°-G.M. Lancisi-G. Salesi, Ancona, Italy; 3 Laboratorio di Microbiologia, Azienda Ospedaliero Universitaria, Ospedali Riuniti Umberto I°-G.M. Lancisi-G. Salesi, Ancona, Italy; Louisiana State University, UNITED STATES

## Abstract

**Background:**

Candidemia is a life-threatening fungal infection and it can affect patients of all ages. Characterization of candidemia in the elderly is lacking.

**Methods:**

We performed a retrospective study of adults (≥ 18 years) with candidemia diagnosed in our center in 2010–2015. Demographics, comorbidities, clinical and microbiologic characteristics, antifungal treatment and outcome were compared between older (≤65 years) and younger (>65 years) patients.

**Results:**

Among 302 patients with candidemia identified during the study period, 188 (62%) belonged to the elderly group. Comorbidities were significantly more frequent in older patients and included chronic pulmonary diseases, cardiovascular diseases, diabetes mellitus, and chronic renal failure (*p* ranging from <0.0001 to 0.017). A significantly higher proportion of older patients had septic shock (*p* = 0.040) at the time of candidemia. *Candida albicans* accounted for 53% of isolates and there were no significant differences between patients’ age and *Candida* species. Thirty-day mortality was significantly higher in older (45%) than in younger (28%) patients (*p* = 0.003). Factors associated with a significant higher proportion of death in the elderly included older age (i.e.: old-old), being hospitalized in ICU rather than in other wards, suffering from chronic pulmonary diseases, the presence of septic shock, multiple organ failure, dialysis and being infected with *C*. *glabrata* (*p* ranging from <0.0001 to 0.034). On multivariate analysis septic shock (HR 1.744 [CI95% 1.049–2.898], p = 0.032) and multiple organ failure (HR 2.242 [CI95% 1.070–4.698], p = 0.032) were independently associated with a higher risk of death. The probability of 30-days survival of older patients was significantly reduced when compared to that of younger patients (*p* = 0.005) who did not receive any treatment. In the elderly, there was a trend toward higher MICs for fluconazole/*C*. *albicans*, fluconazole/*C*. *glabrata*, amphotericin B/*C*. *albicans*, and caspofungin/*C*. *glabrata*.

**Conclusions:**

In our study, we found that elderly patients with *Candida* bloodstream infections are characterized by a high mortality rate. In particular, the lack of any antifungal therapy as well as the occurrence of septic shock increased significantly the overall mortality. Additionally, we found that there was a trend of higher MIC for specific drug/*Candida* combination.

## Introduction

The yeast belonging to the genus *Candida* is the major causative agent of fungal infections in hospitalized patients and the global incidence of candidemia has increased more than fivefold in the last decade [1–7]. Although *Candida albicans* remains the most frequently isolated species, geographical differences are emerging in epidemiology between different countries demonstrating a shift towards non-*albicans* species [5–8].

Candidemia represents a major challenge among the healthcare-related infections due to its difficult diagnostic and therapeutic management [1, 2, 4]. Patients with candidemia have serious underlying diseases and prognosis varies according to several factors [1–7]. The crude mortality of candidemia is extremely high ranging from 36% to 63% depending on the patients population considered [1–13]. The elderly population, which is increasing worldwide, is particularly vulnerable to *Candida* infections. This can be due to several factors such as high frequency of comorbidities, aging-related physiological changes, polypharmacy, and high colonization rate [9–13].

As far, few data are available on the epidemiology and outcome of candidemia in the elderly.

Therefore, we performed a retrospective study of adults with candidemia diagnosed in our center in 2010–2015. Demographics, comorbidities, clinical and microbiologic characteristics, antifungal treatment and outcome were compared between older and younger patients.

## Patients and methods

A retrospective observational study of all cases of candidemia was carried out from January 1, 2010 to December 31, 2015 in a single 980-bedded referral University Hospital in Ancona, Italy. A case of *Candida* bloodstream infection (BSI) was defined as a peripheral isolation of *Candida* species from blood culture in a patient with temporally related clinical signs and symptoms of infection.

All *Candida* BSIs were identified through the microbiological laboratory database. Data regarding demographic characteristics and clinical risk factors were collected from the patient’s medical records. Charlson comorbidity index for each patient was also calculated [14]. Appropriate antifungal therapy was considered when an appropriate drug (based on subsequent in vitro susceptibility testing results) with adequate dosage was started within 72 h from the first blood culture performed. Adequate dosage of an antifungal agent was defined according to IDSA 2009 guidelines [15]. Early central venous catheter (CVC) removal was defined a removal of the line within 48 h from drawing blood culture [16]. Mortality was calculated after 7 and 30 days from the occurrence of the episode of *Candida* BSI. To ascertain the outcome, we considered only those patients from which clinical information was included in the regional health surveillance system. *Candida* species were isolated from blood samples using BacT/ALERT (bioMérieux) and identified with standard techniques. Antifungal susceptibility testing was performed using the SensitreYeastOne colorimetric plate (Trek Diagnostic System) and MIC results of fluconazole, amphotericin B and caspofungin were interpreted according to latest species-specific clinical breakpoints (CBPs) as established by the Clinical and Laboratory Standards Institute (CLSI) [17–19]. The three drugs were selected since each of them is the representative of a specific class.

The present research has been performed in accordance with the ethical standards of the 1964 Declaration of Helsinki and its later amendements. The Institutional Review Board of the Azienda Ospedaliero-Universitaria Ospeadali Riuniti Umberto I°-Lancisi-Salesi granted retrospective access to the data without need for individual informed consent. The consent was not given since the data were analyzed anonymously.

For analysis, patients were divided into two groups according to age: older group (>65 years) and younger group (≤65 years). Patients <18 years old were excluded from this study. Quantitative data were shown as the median with interquartile ranges (Q1–Q3). Qualitative variables were expressed as absolute and relative frequencies. Categorical variables were compared using the χ2 test, whereas Mann–Whitney U test or Fisher exact test were applied for continuous variables. Variables with a *p* ≤0.05 at the descriptive analysis were analyzed by Cox regression. Additionally, survival analysis of older *vs* younger patients was performed to estimate the 30-days cumulative survival probability according to either the lack of antifungal therapy or the presence of septic shock *via* Kaplan-Meier curves. Comparisons of curves were evaluated with the log-rank test. All statistical analyses were performed using the statistical package SPSS for Windows v. 20 (SPSS Inc., Chicago, IL, USA). A *p* value of ≤0.05 was considered to represent statistical significance and all statistical tests were two-tailed.

## Results

A total of 302 patients with candidemia were identified during the study period. There were 114 patients aging ≤ 65 years (median/IQR: 57/48-62 years) and 188 patients aging > 65 years (median/IQR: 76/72-80 years).

Demographic and clinical characteristics are reported in Table 1.

**Table 1:** Demographics and clinical characteristics of 302 patients with BSIs due to *Candida* species considered in this study.

Characteristics		Age		
	All patients (n = 302)	≤65 years (n = 114)	>65 years (n = 188)	*p* value ^*a*^
Male sex, *n (%)*	194 (64)	72 (63)	122 (65)	0.760
Ward				
Internal Medicine, *n (%)*	121 (40)	52 (45)	69 (37)	0.296
Surgery, *n (%)*	76 (25)	27 (24)	49 (26)	
Intensive Care Unit, *n (%)*	105 (35)	35 (31)	70 (37)	
Chronic pulmonary diseases, *n (%)* ^*b*^	47 (16)	3 (1)	44 (23)	<0.0001
Haematological malignancy, *n (%)*	10 (3)	3 (3)	7 (4)	0.607
Cardiovascular diseases, *n (%)* ^*c*^	162 (54)	37 (32)	125 (66)	<0.0001
Neurological diseases, *n (%)* ^*d*^	49 (16)	23 (20)	26 (14)	0.147
Gastrointestinal diseases, *n (%)* ^*e*^	85 (28)	35 (31)	50 (27)	0.441
Diabetes mellitus, *n (%)*	57 (19)	11 (10)	46 (24)	0.001
Solid tumors, *n (%)*	96 (32)	32 (28)	63 (34)	0.323
Chronic renal failure, n (%)	39 (13)	8 (7)	31 (16)	0.017
Charlson comorbidity index, median (IQR) ^*f*^	6 (5–7)	4 (3–6)	7 (5–8)	<0.0001
Previous surgery (<30 days), *n (%)*	160 (53)	61 (54)	99 (53)	0.886
Central venous catheter, *n (%)*	267 (88)	100 (88)	167 (89)	0.770
Central venous catheter-related BSIs, *n (%)* ^*g*^	90 (30)	35 (31)	55 (29)	0.789
Central venous catheter-related BSIs with concomitant bacteriemia, *n (%)*	34 (11)	13 (11)	21 (11)	0.950
Early central venous catheter removal, *n (%)* ^*h*^	155 (58)	65 (65)	90 (54)	0.075
Other devices, *n (%)* ^*i*^	275 (91)	102 (89)	173 (92)	0.451
Multiple organ failure, *n (%)* ^*j*^	18 (6)	8 (7)	10 (5)	0.545
Dialysis, *n (%)*	23 (8)	10 (9)	13 (7)	0.555
Mechanical ventilation, *n (%)*	17 (6)	7 (6)	10 (5)	0.764
Previous invasive procedures (<72 hours), *n (%)* ^*j*^^*k*^	96 (32)	37 (32)	59 (31)	0.846
Parenteral nutrition, *n (%)*	208 (69)	73 (64)	135 (72)	0.157
Steroid therapy, n (%)	82 (27)	26 (23)	56 (30)	0.186
Immunosuppressive therapy, *n (%)* ^*k*^^*l*^	27 (9)	16 (14)	11 (7)	0.015
Neutropenia, *n (%)*	5 (2)	2 (2)	3 (2)	0.916
Septic shock, *n (%)*	36 (12)	8 (7)	28 (15)	0.040
*Candida* species				
*Candida albicans*, *n (%)*	160 (53)	63 (55)	97 (52)	0.874
*Candida parapsilosis*, *n (%)*	69 (23)	24 (21)	45 (24)	
*Candida tropicalis*, *n (%)*	33 (11)	14 (12)	19 (10)	
*Candida glabrata*, *n (%)*	28 (9)	9 (8)	19 (10)	
Other *Candida* species, *n (%)* ^*l*^^*m*^	12 (4)	4 (4)	8 (4)	
Appropriate antifungal therapy, *n (%)* ^*m*^^*n*^	157 (52)	60 (54)	97 (51)	0.861
Primary antifungal therapy				
Azoles, *n (%)*	156 (68)	55 (64)	101 (70)	0.360
Echinocandins, *n (%)*	69 (30)	30 (35)	39 (27)	
Polyenes, *n (%)*	5 (2)	1 (1)	4 (3)	
No treatment, *n (%)*	72 (24)	28 (25)	44 (23)	0.819
Early mortality, *n (%)* ^*n*^^*o*^	75 (25)	23 (20)	52 (28)	0.144
Late mortality, *n (%)* ^*o*^^*p*^	42 (14)	9 (8)	33 (17)	0.006
Overall mortality, *n (%)*	117 (39)	32 (28)	85 (45)	0.003

^*a*^ Comparisons between groups were performed using Chi-Square test or Fisher Exact Test when expected frequencies were less than five.

^*b*^ Chronic pulmonary diseases include asthma, chronic bronchitis, emphysema and lung fibrosis.

^*c*^ Cardiovascular diseases include heart failure, ischemic heart disease, endocarditis and arrhythmia.

^*d*^ Neurological diseases include Parkinson’s disease, Alzheimer’s disease and paralysis.

^*e*^ Gastrointestinal diseases include Crohn’s disease, ulcerative colitis, chronic pancreatitis and gallbladder stones.

^*f*^ IQR, Interquartile range.

^*g*^ A catheter-related candidemia was defined according to the guidelines of the infectious diseases society of America (IDSA: *Mermel LA et al*., *Clinical practice guidelines for the diagnosis and management of intravascular catheter-related infection*: *2009 update by the Infectious Diseases Society of America Clin*. *Infect*. *Dis*. *2009; 49*:*1–45*).

^*h*^ Early central venous catheter removal was considered occurring within 48 h from blood cultures drawing.

^*i*^ Other devices include urinary catheter, surgical drainage, cutaneous gastrostomy and tracheostomy tube.

^*j*^ Multiple organ failure was defined as altered organ function in acutely ill patients involving two or more organ systems (*Irwin RS and Rippe JM*, *Intensive Care Medicine*, *7*^*th*^ *Ed*., *2011*). Among younger patients there were: kidney and cardiovascular failures (n = 3), kidney and gastrointestinal [liver failure and gastrointestinal bleeding] failures (n = 2), respiratory and gastrointestinal failures (n = 2), respiratory and kidney and gastrointestinal failures (n = 1); among older patients there were: kidney and cardiovascular failures (n = 2), kidney and gastrointestinal [liver failure and gastrointestinal bleeding] failures (n = 2), respiratory and neurologic failures (n = 2), respiratory and cardiovascular failures (n = 1), respiratory and kidney failures (n = 1), kidney and neurologic failures (n = 1), respiratory and kidney and cardiovascular failures (n = 1).

^*k*^ Previous invasive procedures include endoscopy and positioning of any device.

^*l*^ Immunosuppressive therapy include calcineurin inhibitors and monoclonal antibodies.

^*m*^ Other *Candida* species included *Candida guilliermondii* (n = 5), *Candida krusei* (n = 2), *Candida lusitaniae* (n = 2), *Candida dubliniensis* (n = 1), *Candida pelliculosa* (n = 1), and *Candida utilis* (n = 1).

^*n*^ Appropriate antifungal therapy was considered when the appropriate drug with adequate dosage was started within 72 hours the first blood culture performed.

^*o*^ Early mortality was considered as occurring within 7 days from the onset of candidemia.

^*p*^ Late mortality was considered as occurring within 30 day from the onset of candidemia.

The majority of patients were hospitalized in medical wards (40%) followed by ICUs (35%) and surgical wards (25%). There were no significant differences between patients age and hospitalization wards. The following comorbidities were significantly more frequent in older patients: higher comorbidity index chronic pulmonary diseases (*p* <0.0001), cardiovascular diseases (*p* <0.0001), diabetes mellitus (*p* = 0.001), and chronic renal failure (*p* = 0.017). Comorbidity score (i.e.: Charlson comorbidity index) was significantly higher in older patients (*p* <0.0001). A significantly higher proportion of older patients had septic shock (*p* = 0.040) at the time of candidemia.

The majority of patients were infected with *C*. *albicans* (53%), followed by *C*. *parapsilosis* (23%), *C*. *tropicalis* (11%), *C*. *glabrata* (9%), and other *Candida* species (4%). There were no significant differences between patients age and *Candida* species.

Primary antifungal therapy consisted of azoles in the majority of patients (68%) followed by echinocandins (30%) and polyenes (2%). Of note, 72 patients (24%) did not receive any treatment. Overall mortality was 39% and it was significantly higher in older (85/188 [45%]) than in younger (32/114 [28%]) patients (*p* = 0.003).

Factors related to outcome of older patients are reported in Table 2.

**Table 2:** Outcome of 188 elderly patients with BSIs due to *Candida* species considered in this study.

Characteristics		30-day outcome	
	Survival (n = 103)	Death (n = 85)	*p* value ^*a*^
Age (years), median (IQR) ^*b*^	76 (72–80)	78 (73–81)	0.017
Male sex, *n (%)*	63(61)	59 (69)	0.238
Ward			
Internal Medicine, *n (%)*	38 (37)	31 (36)	<0.0001
Surgery, *n (%)*	40 (39)	9 (11)	
Intensive Care Unit, *n (%)*	25 (24)	45 (53)	
Chronic pulmonary diseases, *n (%)* ^*c*^	18 (17)	26 (31)	0.034
Haematological malignancy, *n (%)*	4 (4)	3 (4)	1.000
Cardiovascular diseases, *n (%)* ^*d*^	64 (62)	61 (72)	0.163
Neurological diseases, *n (%)* ^*e*^	12 (12)	14 (16)	0.340
Gastrointestinal diseases, *n (%)* ^*f*^	26 (25)	24 (28)	0.643
Diabetes mellitus, *n (%)*	23 (22)	23 (27)	0.452
Solid tumors, *n (%)*	32 (31)	31 (36)	0.434
Chronic renal failure, *n (%)*	13 (13)	18 (21)	0.115
Charlson comorbidity index, median (IQR) ^*b*^	6 (5–7)	7 (6–8)	<0.0001
Previous surgery (<30 days), *n (%)*	57 (55)	42 (49)	0.417
Central venous catheter, *n (%)*	92 (89)	75 (88)	0.814
Central venous catheter-related BSIs, *n (%)* ^*g*^	32 (31)	23 (27)	0.547
Central venous catheter-related BSIs with concomitant bacteriemia, *n (%)*	13 (13)	8 (9)	0.486
Early central venous catheter removal, *n (%)* ^*h*^	48 (47)	35 (41)	0.455
Other devices, *n (%)* ^*i*^	93 (90)	80 (94)	0.335
Multiple organ failure, *n (%)* ^*j*^	1 (1)	9 (11)	0.005
Dialysis, *n (%)*	3 (3)	10 (12)	0.021
Mechanical ventilation, *n (%)*	3 (3)	7 (8)	0.189
Previous invasive procedures (<72 hours), *n (%)* ^*k*^	27 (26)	32 (37)	0.092
Parenteral nutrition, *n (%)*	73 (71)	62 (73)	0.753
Steroid therapy, *n (%)*	26 (25)	30 (35)	0.151
Immunosuppressive therapy, *n (%)* ^*l*^	8 (8)	3 (4)	0.350
Neutropenia, *n (%)*	1 (1)	2 (2)	0.590
Septic shock, *n (%)*	6 (6)	22 (26)	<0.0001
*Candida* species			
*Candida albicans*, *n (%)*	49 (47)	48 (57)	0.029
*Candida parapsilosis*, *n (%)*	28 (27)	17 (20)	
*Candida tropicalis*, *n (%)*	13 (13)	6 (7)	
*Candida glabrata*, *n (%)*	6 (6)	13 (15)	
Other *Candida* species, *n (%)* ^*m*^	7 (7)	1 (1)	
Appropriate antifungal therapy ^*n*^	57 (55)	40 (47)	0.258
Primary antifungal therapy			
Azoles, *n (%)*	62 (74)	39 (65)	0.312
Echinocandins, *n (%)*	19 (22)	20 (33)	
Polyenes, *n (%)*	3 (4)	1 (2)	
No treatment, *n (%)*	19 (18)	25 (29)	0.077

^*a*^ Comparisons between groups were performed using Mann-Whitney test for quantitative variables and Chi-Square test (or Fisher Exact Test when expected frequencies were less than five) for qualitative variables.

^*b*^ IQR, Interquartile range.

^*c*^ Chronic pulmonary diseases include asthma, chronic bronchitis, emphysema and lung fibrosis.

^*d*^ Cardiovascular diseases include heart failure, ischemic heart disease, endocarditis and arrhythmia.

^*e*^ Neurological diseases include Parkinson’s disease, Alzheimer’s disease and paralysis.

^*f*^ Gastrointestinal diseases include Crohn’s disease, ulcerative colitis, chronic pancreatitis and gallbladder stones.

^*g*^ A catheter-related candidemia was defined according to the guidelines of the infectious diseases society of America (IDSA: *Mermel LA et al*., *Clinical practice guidelines for the diagnosis and management of intravascular catheter-related infection*: *2009 update by the Infectious Diseases Society of America Clin*. *Infect*. *Dis*. *2009; 49*:*1–45*).

^*h*^ Early central venous catheter removal was considered occurring within 48 h from blood cultures drawing.

^*i*^ Other devices include urinary catheter, surgical drainage, cutaneous gastrostomy and tracheostomy tube.

^*j*^ Multiple organ failure was defined as altered organ function in acutely ill patients involving two or more organ systems (*Irwin RS and Rippe JM*, *Intensive Care Medicine*, *7*^*th*^ *Ed*., *2011*). There were: kidney and cardiovascular failures (n = 2), kidney and gastrointestinal [liver failure and gastrointestinal bleeding] failures (n = 2), respiratory and neurologic failures (n = 2), respiratory and cardiovascular failures (n = 1), respiratory and kidney failures (n = 1), kidney and neurologic failures (n = 1), respiratory and kidney and cardiovascular failures (n = 1).

^*k*^ Previous invasive procedures include endoscopy and positioning of any device.

^*l*^ Immunosuppressive therapy include corticosteroids, calcineurin inhibitors and monoclonal antibodies.

^*m*^ Other *Candida* species included *Candida guilliermondii* (n = 4), *Candida krusei* (n = 1), *Candida lusitaniae* (n = 2), and *Candida utilis* (n = 1).

^*n*^ Appropriate antifungal therapy was considered when the appropriate drug with adequate dosage was started within 72 hours the first blood culture performed.

The specific risk factors significantly more common among older patients who died within 30 days were: older age (*p* = 0.017), being hospitalized in ICU rather than in other wards (*p* <0.0001), suffering from chronic pulmonary diseases (*p* = 0.034), higher comorbidity index (p <0.0001), the presence of septic shock (*p* <0.0001), multiple organ failure (*p* = 0.005), dialysis (*p* = 0.021), and being infected with *C*. *glabrata* (*p* = 0.029). The following variables were included in the Cox regression analysis: age, ward, the presence of chronic pulmonary disease, comorbidity index, septic shock, dialysis, and *Candida* species. On multivariate analysis septic shock (HR 1.744 [CI95% 1.049–2.898], *p* = 0.032) and multiple organ failure (HR 2.242 [CI95% 1.070–4.698], *p* = 0.032) were independently associated with a higher risk of death. Factors related to outcome of younger patients are reported in Table 3.

**Table 3:** Outcome of 114 younger patients with BSIs due to *Candida* species considered in this study.

Characteristics		30-day outcome	
	Survival (n = 82)	Death (n = 32)	*p* value ^*a*^
Age (years), median (IQR) ^*b*^	57 (44–61)	58 (51–63)	0.067
Male sex, *n (%)*	48 (59)	32 (75)	0.101
Ward			
Internal Medicine, *n (%)*	40 (49)	12 (38)	0.168
Surgery, *n (%)*	21 (26)	6 (19)	
Intensive Care Unit, *n (%)*	21 (26)	14 (44)	
Chronic pulmonary diseases, *n (%)* ^*c*^	1 (1)	2 (6)	0.189
Haematological malignancy, *n (%)*	1 (1)	2 (6)	0.189
Cardiovascular diseases, *n (%)* ^*d*^	28 (34)	9 (28)	0.537
Neurological diseases, *n (%)* ^*e*^	18 (22)	5 (16)	0.605
Gastrointestinal diseases, *n (%)* ^*f*^	26 (32)	9 (28)	0.709
Diabetes mellitus, *n (%)*	6 (7)	5 (16)	0.286
Solid tumors, *n (%)*	21 (26)	11 (34)	0.349
Chronic renal failure, *n (%)*	6 (7)	2 (6)	1.000
Charlson comorbidity index, median (IQR) ^*b*^	4 (3–5)	5 (4–6)	<0.0001
Previous surgery (<30 days), *n (%)*	45 (55)	16 (50)	0.638
Central venous catheter, *n (%)*	71 (87)	29 (91)	0.554
Central venous catheter-related BSIs, *n (%)* ^*g*^	28 (34)	7 (22)	0.260
Central venous catheter-related BSIs with concomitant bacteriemia, *n (%)*	11 (13)	2 (6)	0.279
Early central venous catheter removal, *n (%)* ^*h*^	40 (49)	16 (50)	0.906
Other devices, *n (%)* ^*i*^	73 (89)	29 (90)	0.860
Multiple organ failure, *n (%)* ^*j*^	6 (7)	2 (6)	1.000
Dialysis, *n (%)*	6 (7)	4 (1)	0.463
Mechanical ventilation, *n (%)*	3 (4)	4 (1)	0.095
Previous invasive procedures (<72 hours), *n (%)* ^*k*^	25 (30)	12 (38)	0.472
Parenteral nutrition, *n (%)*	47 (57)	26 (81)	0.016
Steroid therapy, *n (%)*	22 (27)	4 (13)	0.137
Immunosuppressive therapy, *n (%)* ^*l*^	12 (15)	4 (13)	1.000
Neutropenia, *n (%)*	0 (0)	2 (6)	0.077
Septic shock, *n (%)*	1 (1)	7 (22)	0.0005
*Candida* species			
*Candida albicans*, *n (%)*	42 (51)	21 (66)	0.418
*Candida parapsilosis*, *n (%)*	19 (23)	5 (16)	
*Candida tropicalis*, *n (%)*	12 (15)	2 (6)	
*Candida glabrata*, *n (%)*	7 (9)	2 (6)	
Other *Candida* species, *n (%)* ^*m*^	2 (2)	2 (6)	
Appropriate antifungal therapy ^*n*^	43 (52)	17 (53)	0.947
Primary antifungal therapy			
Azoles, *n (%)*	38 (63)	17 (65)	0.800
Echinocandins, *n (%)*	21 (35)	9 (35)	
Polyenes, *n (%)*	1 (2)	0 (0)	
No treatment, *n (%)*	22 (27)	6 (19)	0.367

^*a*^ Comparisons between groups were performed using Mann-Whitney test for quantitative variables and Chi-Square test (or Fisher Exact Test when expected frequencies were less than five) for qualitative variables.

^*b*^ IQR, Interquartile range.

^*c*^ Chronic pulmonary diseases include asthma, chronic bronchitis, emphysema and lung fibrosis.

^*d*^ Cardiovascular diseases include heart failure, ischemic heart disease, endocarditis and arrhythmia.

^*e*^ Neurological diseases include Parkinson’s disease, Alzheimer’s disease and paralysis.

^*f*^ Gastrointestinal diseases include Crohn’s disease, ulcerative colitis, chronic pancreatitis and gallbladder stones.

^*g*^ A catheter-related candidemia was defined according to the guidelines of the infectious diseases society of America (IDSA: *Mermel LA et al*., *Clinical practice guidelines for the diagnosis and management of intravascular catheter-related infection*: *2009 update by the Infectious Diseases Society of America Clin*. *Infect*. *Dis*. *2009; 49*:*1–45*).

^*h*^ Early central venous catheter removal was considered occurring within 48 h from blood cultures drawing.

^*i*^ Other devices include urinary catheter, surgical drainage, cutaneous gastrostomy and tracheostomy tube.

^*j*^ Multiple organ failure was defined as altered organ function in acutely ill patients involving two or more organ systems (*Irwin RS and Rippe JM*, *Intensive Care Medicine*, *7*^*th*^ *Ed*., *2011*). There were: kidney and cardiovascular failures (n = 3), kidney and gastrointestinal [liver failure and gastrointestinal bleeding] failures (n = 2), respiratory and gastrointestinal failures (n = 2), respiratory and kidney and gastrointestinal failures (n = 1).

^*k*^ Previous invasive procedures include endoscopy and positioning of any device.

^*l*^ Immunosuppressive therapy include corticosteroids, calcineurin inhibitors and monoclonal antibodies.

^*m*^ Other *Candida* species included *Candida dubliniensis* (n = 1), *Candida guilliermondii* (n = 1), *Candida krusei* (n = 1), and *Candida pelliculosa* (n = 1).

^*n*^ Appropriate antifungal therapy was considered when the appropriate drug with adequate dosage was started within 72 hours the first blood culture performed.

The specific risk factors significantly more common among younger patients who died within 30 days were: higher comorbidity index (*p* < 0.0001), parenteral nutrition (*p* = 0.016) and the presence of septic shock (*p* = 0.0005). These variables were included in the Cox regression analysis. Again, only the presence of septic shock resulted independently associated with a significant higher risk of death (HR 3.304 [CI95% 1.422–7.677], *p* = 0.005). Because septic shock had such profound adverse prognostic impact on candidemia regardless patients’ age, we analyzed the occurrence of septic shock in patients according to appropriate therapy (9% [14/157]), no appropriate therapy (18% [13/73]), and no therapy (13% [9/72] and we did not find any statistical difference (*p* = 0.150).

**Fig 1:**
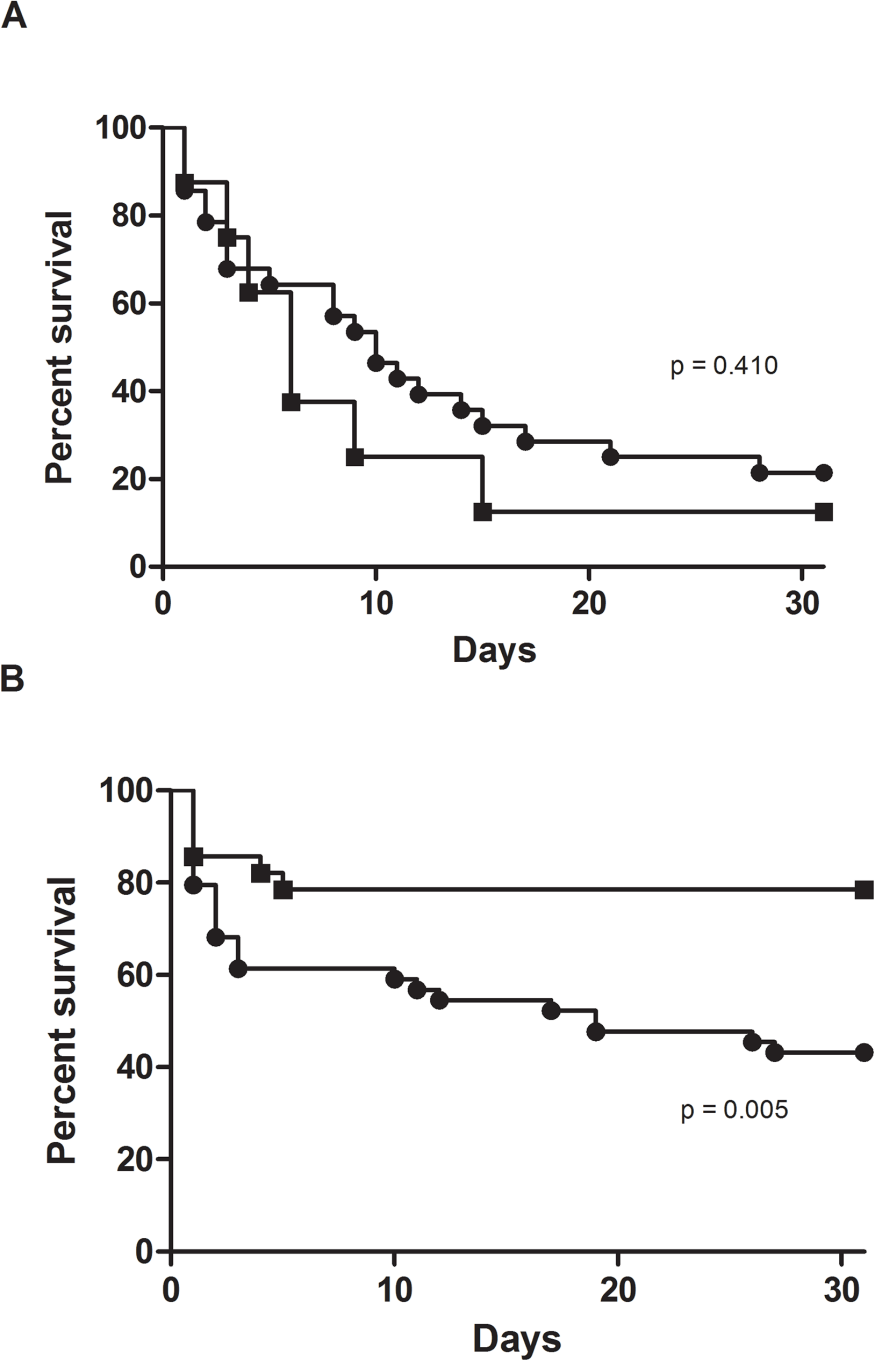
30-days survival of older (circle) *vs* younger (squares) patients with candidemia with septic shock (A) and without any antifungal therapy (B).

We further analyzed the probability of 30-days survival of older *vs* younger patients suffering from septic shock and we did not find any statistically significance (Fig 1A; *p* = 0.410).

Since there was a high proportion of candidemic patients who did not received any treatment, we analyzed the impact of lack of antifungal therapy on survival. As shown in Fig 1B, the probability of 30-days survival of older patients was significantly reduced when compared to that of younger patients (*p* = 0.005) who did not receive any treatment.

**Fig 2:**
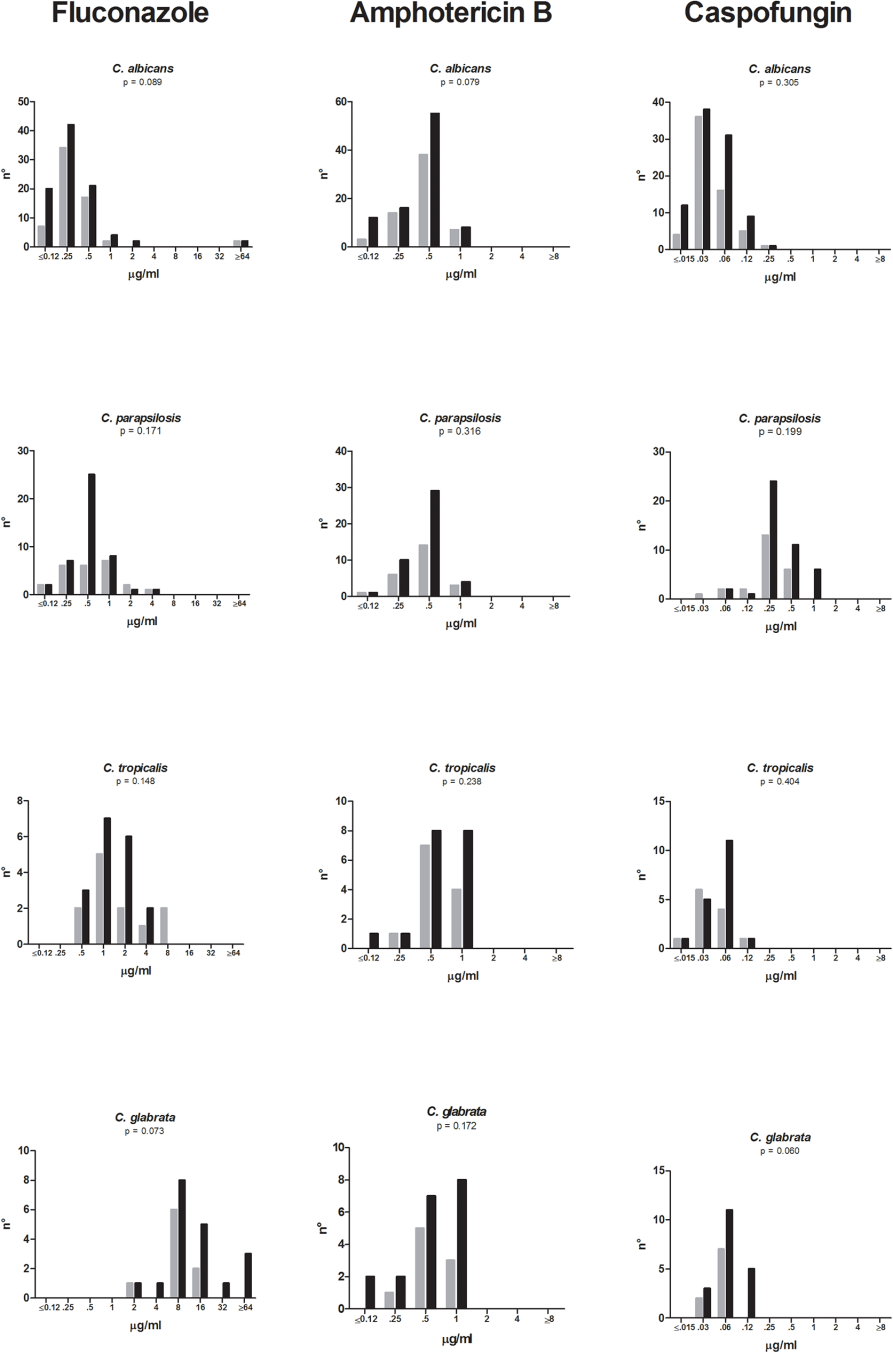
Fluconazole (A), amphotericin B (B), and caspofungin (C) MICs for clinical isolates of *Candida* spp. recovered from younger (gray bars) and older (black bars) patients.

Fig 2 depicts antifungal susceptibility patterns of fluconazole, caspofungin and amphotericin B against the isolates belonging to the four most common *Candida* species. MICs results were available for 96% of the isolated yeasts. Fluconazole resistance accounted for 3%, 6%, 11%, and 0% of isolates of *C*. *albicans* (4/160), *C*. *tropicalis* (2/33), *C*. *glabrata* (3/28), and *C*. *parapsilosis* (0/69), respectively. All isolates were susceptible to amphotericin B (WT phenotype) and to caspofungin. Although in the elderly there was a trend toward higher MICs for fluconazole/*C*. *albicans*, fluconazole/*C*. *glabrata*, amphotericin B/*C*. *albicans*, and caspofungin/*C*. *glabrata*, a statistically significant difference was not reached. There were 21/188 (11%) of older patients *vs* 9/114 (8%) of younger patients exposed to fluconazole before developing candidemia (*p* = 0.356).

## Discussion

In order to investigate the outcome predictors of candidemia in the elderly, we evaluated a database of patients with candidemia diagnosed over a six years period in our hospital and compared clinical and microbiologic characteristics in elderly *versus* younger patients. Data from the literature often report older age as an independent factor for mortality in patients with candidemia [2, 5–7, 9, 11, 12]. In our cohort, elderly patients (>65 years) represented 62% of the entire study group (188/302). This proportion, which is somewhat higher than those reported by other studies, makes our population particularly suitable to be investigated in this regard.

In our study, we found that elderly patients with *Candida* bloodstream infections are characterized by a high mortality rate. In particular, the lack of any antifungal therapy as well as the occurrence of septic shock increased significantly the overall mortality. Additionally, we found that there was a trend of higher MIC for specific drug/*Candida* combination.

We confirmed that the overall mortality in the elderly was significantly higher than that observed in younger patients (45% *vs* 28%). Similarly, two recent studies showed that 30-day mortality increased from 17% to 32% and from 45% to 70% in younger and older patients, respectively [9, 11]. This wide variation of mortality among studies can be due to several reasons such as the definition of elderly, the proportion of immunocompromised patients included (i.e.: hematologic), the type of antifungal utilized for primary therapy, the interval between blood drawing and initiation of drug, the variable CVC management. In particular, in our series there were only 10 hematologic patients (3 in younger and 7 in older groups) and neutropenia was found only in five patients.

As expected, we found a significant higher proportion of older patients suffering from specific comorbidities, such as chronic pulmonary diseases, diabetes mellitus, chronic renal failure and cardiovascular diseases. While the occurrence of the first three types of diseases was consistent with that reported in the literature (ranging from 16% to 24%), cardiovascular diseases accounted for 66% of our older patients. This figure is higher than that observed in other studies as far [9, 11]. Although septic shock was significantly more frequent in elderly than younger patients, its occurrence resulted to be an independent factor for mortality in both groups [20, 21]. Interestingly, we found that multiple organ failure (i.e.: altered organ function in acutely ill patients involving two or more organ systems) was an independent factor for higher risk of mortality only in older patients.

Although we found that timely antifungal therapy and early source control (i.e., removal of central lines), were not significantly associated with a better survival, both characteristics were proportionally higher in elderly patients who survived.

As reported in other studies, we found a high proportion of patients not receiving any antifungal drug (24% in our series) [11]. To see whether the lack of antifungal therapy would affect the prognosis of candidemia in the elderly, we performed Kaplan Meyer analysis and showed that there was a significantly higher proportion of elderly patients no treated who died within 30 days with respect to younger group. Although it’s hard to explain why a high proportion of patients in our study was not treated for *Candida* infection, we do exclude that this can be due by a decision of family / doctor / organizational factor, since the Italian health system provides any kind of therapeutic approach regardless of patients’ age thereby making any comparison with the results of other studies very difficult [11].

Unlike other reports, we did not find any difference between patients’ age and *Candida* species, thus reinforcing the importance of knowing the local epidemiology. Several studies from United States and Europe have recently demonstrated that older patients have increased risk of candidemia due to *C*. *glabrata* [1, 4, 8, 22, 23]. In contrast, an association between *C*. *tropicalis* candidemia and elderly patients has been reported in Brazil, Saudi Arabia, Taiwan and Singapore [24–27]. The most common isolated *Candida* species found in our study were represented by *C*. *albicans* followed by *C*. *parapsilosis*, regardless of patients’ age. This rank order is in line with that previously reported in studies of candidemia conducted in Italy [6, 7, 12]. Previous exposure to azoles or high proportion of specific underlying diseases, like malignancies, might explain these microbiologic differences.

It’s interesting to note that in our old patients group infections due to *C*. *glabrata* were characterized by a significant higher proportion of death with respect to other *Candida* species. This characteristic, however, was lost in multivariate analysis.

In this study, we also evaluated in vitro susceptibility patterns of fluconazole, amphotericin B and caspofungin against our collection of *Candida* clinical isolates. The three drugs were selected since each of them is the representative of a specific class. First, we found that occurrence of antifungal resistance was found only for fluconazole *vs C*. *albicans* (3%), *C*. *tropicalis* (6%), and *C*. *glabrata* (10%). Second, we observed a trend towards higher MICs for specific combinations drug/*Candida* strains isolated from the elderly. This was particularly evident, although not statistically significant, for fluconazole. This finding can be due to the fact that elderly patients, being more susceptible to various infections, are often exposed to several antibiotics and antifungal agents thus determining a selection of less susceptible isolates [10]. In reviewing our database we found that 11% (21/188) of older patients *vs* 8% (9/114) of younger patients were exposed to fluconazole before developing candidemia. Of note, we observed a shift to higher caspofungin MICs for *C*. *glabrata* isolates from the elderly. Although this finding again was not significant and the overall number of isolates was quite low (9/younger, 19/older), this phenomenon deserves not to be underestimated. Lately, an increase of reduced susceptibility to echinocandins among fluconazole-resistant isolates of *C*. *glabrata* has been documented [28, 29].

The present study have some limitations. First, being a retrospective study encompassing several departments and medical disciplines over a six years period, there was not a univocal management of each individual case. Second, although we have made every attempt to collect and analyze as many as clinical data as possible to reveal useful information for the patients management, some variables could not be explored because of missing data. Finally, since our data come from a single center experience, our findings may not be relevant to other patients populations.

In conclusion, candidemia in the elderly has become an important clinical problem accounting for a great proportion of patients with this invasive fungal disease. Since it is characterized by a poor prognosis, the identification of host- (i.e.: specific comorbidities) and healthcare-related risk factors (i.e.: lack of antifungal therapy) are crucial to prevent a negative outcome.
